# Does valence in the visual domain influence the spatial attention after auditory deviants? Exploratory data

**DOI:** 10.3389/fnbeh.2013.00006

**Published:** 2013-02-15

**Authors:** Lisa Schock, Saurabh Bhavsar, Liliana R. Demenescu, Walter Sturm, Klaus Mathiak

**Affiliations:** ^1^Department of Psychiatry, Psychotherapy and Psychosomatics, Medical School, RWTH Aachen UniversityAachen, Germany; ^2^Interdisciplinary Centre for Clinical Research, Medical School, RWTH Aachen UniversityAachen, Germany; ^3^Jülich-Aachen Research Alliance-Translational Brain MedicineJülich, Aachen, Germany; ^4^Department of Neurology, Clinical Neuropsychology, Medical School, RWTH Aachen UniversityAachen, Germany; ^5^Institute for Neuroscience and Medicine, INM-1, Research Centre JülichJülich, Germany

**Keywords:** auditory deviants, dichotic listening, spatial attention, valence, multisensory perception

## Abstract

The auditory mismatch responses are elicited in absence of directed attention but are thought to reflect attention modulating effects. Little is known however, if the deviants in a stream of standards are specifically directing attention across modalities and how they interact with other attention directing signals such as emotions. We applied the well-established paradigm of left- or right-lateralized deviant syllables within a dichotic listening design. In a simple target detection paradigm with lateralized visual stimuli, we hypothesized that responses to visual stimuli would be speeded after ignored auditory deviants on the same side. Moreover, stimuli with negative valence in the visual domain could be expected to reduce this effect due to attention capture for this emotion, resulting in speeded responses to visual stimuli even when attention was directed to the opposite side by the auditory deviant beforehand. Reaction times of 17 subjects confirmed the speeding of responses after deviant events. However, reduced facilitation was observed for positive targets at the left after incongruent deviants, i.e., at the right ear. In particular, significant interactions of valence and visual field and of valence and spatial congruency emerged. Pre-attentive auditory processing may modulate attention in a spatially selective way. However, negative valence processing in the right hemisphere may override this effect. Resource allocation such as spatial attention is regulated dynamically by multimodal and emotion information processing.

## Introduction

Spatial attention distribution serves to enable an efficient interaction with the environment. The importance of such a mechanism is reflected in the huge impact of impairments of attention in the left visual field—unilateral neglect—on simple activities of daily living in patients with right-hemisphere damage (for a review see Danckert and Ferber, 2006). Valid spatial cueing of behaviorally relevant target stimuli can facilitate reaction to these targets; invalid cueing can enhance reaction times (Posner, 1980). Previous studies distinguished between exogenous and endogenous—stimulus-driven and goal-directed—attention (for a review see Bartolomeo and Chokron, 2002). Exogenous cues trigger stimulus-driven shifts of attention when appearing in the left or right hemispace, even if cue and target stimuli are presented in different modalities (e.g., Eimer and Driver, 2001).

Another important factor concerning the distribution of attention in space is the salience of stimuli. Emotional valence of stimuli can influence attention-related processing (Eastwood et al., 2001; Fenske and Eastwood, 2003). Negative valence seems to serve as an attention “magnet”; it can narrow the attention focus accompanied by enhanced processing of the negative stimuli (Fenske and Eastwood, 2003). In visual search, negative stimuli are found faster and with lesser errors than positive stimuli (Eastwood et al., 2001). Stimuli with negative valence can even overcome neglect symptoms in the left visual field in patients with right-hemisphere damage (Grabowska et al., 2011). On the other hand, it was shown that negative emotionally arousing stimuli interfere with processing of left visual field targets when competing for attention resources (Hartikainen et al., 2007). A neural network of limbic regions like amygdala and insula with the anterior cingulate cortex is known to play a role in salience detection and evaluation (for a review see Seeley et al., 2007; Menon and Uddin, 2010; Santos et al., 2011).

Deviant acoustic stimuli in a sequence of rapidly presented standard stimuli elicit automatic mismatch responses in auditory cortices measured by electrophysiological as well as neuroimaging methods (EEG: Näätänen et al., 1989; fMRI: Mathiak et al., 2002). In spite of the pre-attentive nature of these responses, stimulus-driven attention shifts to the side of the deviant are possible (Näätänen, 1995; Schröger, 1996). In one of few studies on behavioral equivalence of mismatch processes, Schröger (1996) used a dichotic listening design with two different stimulus streams on both ears and instructed participants to ignore one ear while they had to do a GoNogo-task on the other ear. It was demonstrated that reaction time costs occurred if the target stimulus of the GoNogo-task was preceded by a deviant on the to-be-ignored ear. The authors concluded that deviants in an oddball design elicited a shift of attention within the auditory domain.

The goal of the present study was to demonstrate spatial shifts in visual attention by lateralized auditory deviants in an unattended oddball design. Visual stimuli of positive and negative valence in the left or right visual field represented target stimuli, which were either spatially congruent or incongruent with the deviating dichotic left-ear or right-ear stimuli. An attention shift to the side of the deviant was hypothesized, resulting in response acceleration to spatially congruent visual stimuli. Negative stimuli were expected to reduce reaction time costs in incongruent trials. Therefore, the ANOVA of reaction times was expected to reveal an effect of spatial congruency and more importantly an interaction between congruency and valence.

## Materials and methods

### Subjects

Seventeen healthy volunteers (11 females, age 19–43 years, mean age 27.5 ± 6.7 years) participated in the experiment. All subjects were right-handed as indicated by the laterality quotient of the Edinburgh Inventory (Oldfield, 1971). To exclude any psychiatric condition, participants were screened with the Structured Clinical Interview (SCID-I; Wittchen et al., 1997) for the Diagnostic and Statistical Manual of Mental Disorders (DSM-IV). The study was approved by the local Ethics Committee of the Medical School of the RWTH Aachen University and was conducted in accordance with the Code of Ethics of the World Medical Association (Declaration of Helsinki). Informed consent was obtained prior to participation in the study.

### Stimuli and design

In the mismatch design, deviant stimuli in a sequence of standard stimuli are known to be detected even in the absence of directed attention. Therefore, we employed an unattended sequence of stimuli. To obtain an effect on spatial attention, dichotic stimuli were applied and deviance occurred on one side only. On this auditory “background” visual stimuli were presented with a simple detection task. The schematic faces were presented shortly (175 ms) after either a standard or a deviant stimulus. To test spatial attention these were either at the left or at the right side and to test valence effects the schematic face bore either a positive or negative expression.

#### Dichotic stimulation

Processing of left- and right-ear stimuli usually interacts at early processing levels, e.g., due to early processing of inter-aural time and level differences. Therefore, we chose a paradigm, which has been shown to elicit side-specific responses at the level of the auditory cortices (Hertrich et al., 2002). The auditory stimulation was chosen to match the study in Schock et al. (2012), but conducted in an independent sample. Sequences of consonant-vowel (CV) syllables /ba/ and /ka/ were applied dichotically. The /ba/ was constituted of two different versions from the same speaker providing random phase-relation between left- and right-ear. The stimuli were always synchronous over both ears. The deviant events were composed of the syllable /ka/ presented to one ear and the standard syllable to the other ear (/baL/-/ka/ or /ka/-/baR/). Thus, the two cues voice-onset time and place-of-articulation changed facilitating a robust mismatch response over subjects even when stimuli were ignored. The stimuli had the same duration (300 ms) and sound level.

**Figure 1:**
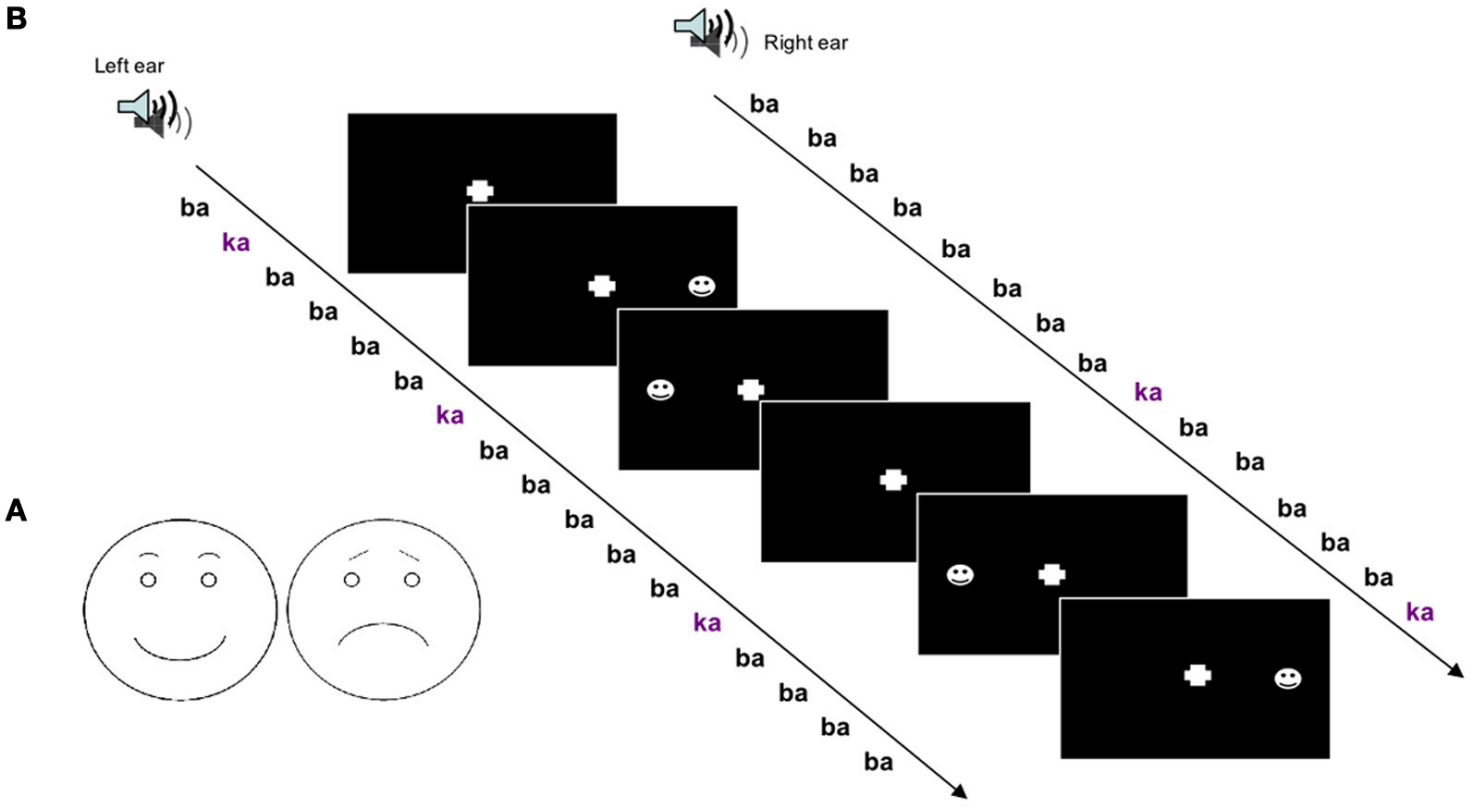
**(A)** Schematic drawings of faces with positive and negative valence were used to investigate crossmodal attention-directing effects of auditory deviants. **(B)** Experimental run; a dichotic oddball paradigm was applied to study crossmodal spatial cueing with acoustic deviants as cues and visual stimuli as targets.

The task-irrelevant oddball sequence with dichotic stimuli was administered over noise-cancellation headphones (HDA 200, Sennheiser, Germany) with a constant stimulus onset asynchrony (SOA) of 500 ms. Sound pressure level was adjusted to comfortable hearing level in a quiet office environment. The resulting level was in the range 65–80 dB SPL. Out of the 10% deviant /ka/ stimuli, half were presented at the left and half at the right side. Deviants were random with different randomization sequences across sessions and subjects. Subjects were instructed to ignore the sounds and only pay attention to the visual stimuli (see Figure 1B).

#### Visual stimulation

For visual stimulation schematic faces were chosen which exhibited either a positive or a negative expression. The positive expression included a half-elliptic mouth shape and round-shaped eyebrows. The negative expression was achieved by inverting the mouth downwards and orienting the inner eyebrow ends upwards (see Figure 1A). Emotional appraisal of the stimuli was validated beforehand. In a pre-study on 10 healthy subjects, the rather unambiguous positive and negative stimuli (see Figure 1A) were rated as happy or sad, respectively, without variance.

Visual stimuli were presented with an SOA of 175 ms after 50% of the deviants with a stimulus duration of 150 ms. Visual stimuli were either of positive or negative valence and appeared either in the left or right visual field, spatially congruent or incongruent with the deviant syllable. Participants were instructed to press a button with the index finger of the right hand as fast as possible at stimulus appearance without distinction of visual field or valence. Subjects were instructed to fixate on a cross in the middle of the screen.

### Procedure

Computing the factors *valence* (positive, negative), *visual field* (left, right), and *spatial congruency* with deviant (left-ear deviant, right-ear deviant), eight (2 × 2 × 2) conditions resulted for acoustic deviants followed by a visual stimulus. As control condition, standard events followed by a visual stimulus were presented as well, providing four different conditions (two 2-level factors *valence* and *visual field*). The experiment comprised 15 stimuli for each of the eight “deviant + face” conditions and 30 stimuli for each of the four “standard + face” conditions to balance the number of stimuli. The design also accounted for a balanced distribution of deviant acoustic stimuli and visual stimuli throughout the oddball sequence with a minimum of two standard syllable pairs (/baL/-/baR/) in between syllable pairs with a deviant (/baL/-/ka/ or /ka/-/baR/). Moreover, a minimum of two “no face trials” was maintained between two “face trials” to prevent an overlap of the button press in response to a visual stimulus and the presentation of the next visual stimulus. In the randomization procedure, first, the auditory oddball design was randomized; second, randomly 15 left and 15 right deviant events were selected and assigned to the “deviant + face” condition. Third, from the standard events one was randomly selected and assigned to the “standard + face” condition only if it maintained at least two-trial distance from any other face trial; this procedure was repeated until 30 trials were successfully assigned to the “standard + face” condition.

Stimulus presentation with ms-precise synchronization between auditory and visual modality and response time recording was conducted using the software Presentation 10.0 (Neurobehavioral Systems, Inc., Albany, CA) using a standard personal computer. The experiment lasted about 20 min for each participant.

### Data analysis

Button presses in response to visual stimuli were analyzed by subtracting onset of the visual stimulus from onset of button press. The mean reaction time value (in ms) was obtained for each participant. The reaction to visual stimuli after standard events was compared to the reaction to visual stimuli after deviant events without considering spatial congruency in order to detect possible alerting effects of the deviant syllables; therefore, responses of the two valence and the two visual field conditions were pooled in the analysis (T_67_). A 2 × 2 × 2 repeated-measures ANOVA with the factors *valence* (positive, negative), *visual field* (left, right), and *spatial congruency* (left-ear deviant, right-ear deviant) was conducted. Subject was the repeating factor. Significance level was set at *p* < 0.05. Paired *t*-tests disentangled the effects *post-hoc*.

## Results

**Figure 2:**
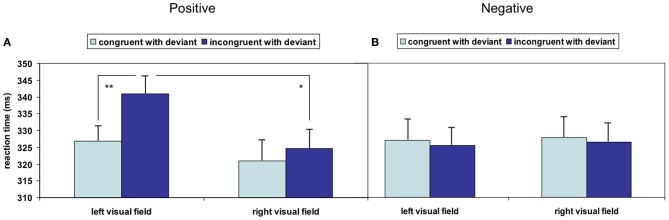
**Significant crossmodal interactions of stimulation sides emerged in the (A) positive valence condition but not in the (B) negative valence condition.** A congruency effect could be detected in the left visual field with positive valence, whereas the detection of stimuli with negative valence was not speeded by cross-modal congruency (^*^p < 0.05; ^**^p < 0.01; mean ± SE).

Overall deviant stimuli had a facilitating effect on responses to succeeding face stimuli as compared to standard stimuli (T_67_ = −5.369, *p* < 0.001); this acceleration was further modulated by stimulus properties: the repeated-measures ANOVA analyzing the influence of the factors *valence* of the visual stimulus, *visual field* and *spatial congruency* with acoustic deviant on reaction time yielded no significant main effect but significant interactions of valence and visual field [*F*_(1, 16)_ = 6.122, *p* = 0.025] as well as valence and spatial congruency [*F*_(1, 16)_ = 9.595, *p* = 0.007]. The other double and triple interactions failed significance (all *p* > 0.15). To account for a potentially skewed distribution of the reaction times, the analysis was repeated after square root transformation; no influence of the distribution emerged (valence X visual field: *F*_(1, 16)_ = 6.753, *p* = 0.019; valence X spatial congruency: *F*_(1, 16)_ = 7.728, *p* = 0.013; other *p* > 0.15). Pair-wise comparisons revealed significantly faster responses to positive stimuli in the left visual field after left-ear deviants compared to after right-ear deviants, reflecting a reaction time acceleration due to spatial congruency (T_16_ = −4.024, *p* = 0.001; Figure 2A). Spatial incongruence did not provoke a similar slowing in the right visual field: responses to incongruent positive stimuli in the right visual field were significantly faster than those in the left visual field (T_16_ = 2.338, *p* = 0.033; Figure 2A). No significant effect emerged when stimuli expressed negative valence (Figure 2B). Non-parametric testing confirmed both *post-hoc t*-tests (Wilcoxon-Test: *W* = 141, *p* < 0.001; *W* = 117, *p* < 0.028), which may argue against an outlier effect in the relatively small sample.

## Discussion

In the present study, acoustic deviants in a dichotic oddball paradigm were expected to direct attention across modalities in interaction with emotions. In analogy to the cueing effect of Posner, deviants were hypothesized to serve as crossmodal spatial cues—with positive and negative schematic faces in the left or right visual field as targets. No overall congruency effect emerged, but an interaction of emotional valence and congruency effect emerged. Reactions in response to positive visual stimuli in the left hemifield were slower when deviants had shifted attention to the right beforehand, compared to responses to spatially congruent stimuli. Specifically, visual stimuli expressing negative emotion enabled faster reaction due to attention capture.

### Attention-directing deviants

The mismatch response is a well-established tool in the investigation of neural responses expressing relevance and discrimination of stimulus features (Näätänen and Winkler, 1999). It is thought to measure attention mechanisms without the explicit allocation of attention (Näätänen, 1992). Indeed, our study confirmed that the presentation of a deviant stimulus speeded the responses to visual stimuli. This is in line with previous studies on cueing (see Posner, 1980) and designs comparable to the unattended oddball design (e.g., Sussman et al., 2003). Spatial resource allocation was reflected in designs presenting different stimuli to the left and the right ear. Deviant acoustic stimuli direct attention to one side resulting in slower processing at the other side (Schröger, 1996). We studied whether acoustic deviants are suitable to cue stimuli of another modality in different spatial locations. The cueing paradigm described by Posner (1980) considered valid peripheral cues indicating the location of the target and invalid cues directing attention away from the target location. Time-dependent relation of cue and target is a critical factor to maintain relevance of the cue for the target detection, i.e., below 300 ms (compare Spence and Driver, 1994). We found a spatially selective slowing of the detection of incongruent targets, i.e., positive expressions on the opposite side to the preceding deviant. This confirms that the processing of deviant stimuli—even if unattended—may lead to shifts in supramodal spatial attention.

Previously we have found that mismatch responses to dichotic deviants are modulated by lateralized stimuli in other modalities (Mathiak et al., 2005). The responses to deviants were enhanced by visual stimuli at or toward the same side. In the same vein, tactile congruent stimuli increased responses to auditory events as early as 100 ms after stimulus onset (Menning et al., 2005). Taken together these findings suggest a bidirectional interaction between spatial attention across modalities even in the absence of explicit attention. In these studies, the spatial nature of the stimuli emerged from dichotic presentation considering the left and right auditory channel as equivalent to a spatial location in the left and right hemifield. Therefore, spatial localization or mere hemispheric representation may underlie the observed multisensory interactions.

The audio-visual interaction seems to be strongest at the right hemisphere. We found the incongruence effect in the left visual field only. This is in accordance with the effects of visual stimuli on mismatch responses, which were stronger at the right hemisphere (Mathiak et al., 2005). Considering the face-like target stimuli, this is well in-line with models of right-lateralized face processing (Kanwisher et al., 1997). More general, spatial attention networks were considered lateralized to the right hemisphere (Corbetta and Shulman, 2002). Thus a speeding of responses to visual stimuli may have been expected strongest in the left hemifield. Moreover, this lateralization pattern suggests interaction with right-hemispheric emotion processing as well.

### Interaction with emotions

Models of hemispheric dominance of emotion processing localize positive valence in the left hemisphere and negative valence in the right hemisphere (for a review, see Demaree et al., 2005). Particularly automatic processing of emotions was found to elicit right-lateralized patterns (e.g., Dyck et al., 2011). Mismatch negativity has been found to be reduced by visual stimuli with positive but not negative valence (Surakka et al., 1998). In our study, reaction time costs in response to spatially incongruent stimuli emerged only in the positive valence condition (Figure 2) as well. This may be attributed to low arousal values of sadness. In contrast considering high arousal, positive and negative emotions (happy and angry) ameliorated lateralized attention deficits in hemineglect; Vuilleumier and Schwartz (2001) found schematic face to bias attention to one side more strongly than neutral faces even when conscious processing was impaired. Amygdala and extrastriate cortex were thought to be the underlying neural networks (Vuilleumier et al., 2002). Similar to faces, emotional prosody yielded attention shifts into the affected hemispace (Grandjean et al., 2008). Emotional arousal and spatial attention conceivably interact at early processing stages (see Grabowska et al., 2011).

Negative valence—even if not task-relevant and not attended—may suffice to interfere with a right-hemispheric network that enables crossmodal shifts of attention. Indeed, mood induction may also modulate mismatch responses at the right hemisphere (Schock et al., 2012). Thus, similar to the bidirectional interaction of spatial attention across modalities, bidirectional interaction between spatial attention and negative emotion can be expected. Indeed, altered mood in depression can lead to a bias in visuospatial attention (Liotti and Mayberg, 2001), which may be due to reduced right-hemispheric arousal (Schock et al., 2011). It is rather notable how apparently small context effects can influence crossmodal interactions.

As an alternative to the valence hypothesis, the right-hemisphere theory hypothesizes dominance of the right hemisphere in processing emotions (Borod et al., 1998). Therefore, negative valence may be expected to be unaffected by reaction time costs because of higher emotional salience of the stimuli (Eastwood et al., 2001; Fenske and Eastwood, 2003). The mechanism may not differ completely since the predicted effects on the attention network are similar (Killgore and Yurgelun-Todd, 2007). Indeed, more salient positive stimuli can be expected to reduce the crossmodal interaction influencing detection speed.

### Limitations

One feature of mismatch experiments is that the number of target stimuli is relatively low, particularly if they are distributed between different conditions. Therefore, outliers may have affected the statistical analysis despite corroborating evidence from root-transformed data and non-parametric testing. Extending the test duration much over the 20 min may have unwanted effects on attention and arousal. Näätänen et al. (2004) suggested a variant with interleaved deviants with different auditory features. Such a design however is not feasible with the intervening visual stimuli. Furthermore, the number of subjects in the present experiment (*n* = 17) is not high enough for conclusions on generalizability. For instance the schematic faces were previously applied successfully to study valence effects (e.g., Eastwood et al., 2001) but the generalizability to less abstract depictions or natural faces is unclear (compare Dyck et al., 2010). A number of additional confounders still need to be taken into account such as the type of the auditory stimuli (phonetic or noises), the timing of the stimuli, the level of alertness, and the task (simple detection, localization, or discrimination). Therefore, another limitation lies in the high number of parameters which may influence the level of interaction between the modalities.

## Conclusion

Reaction times confirmed crossmodal spatial cueing effects of auditory deviants in a mismatch design. Deviant stimuli in a task-irrelevant oddball design were shown to serve as attention-directing cues that are effective across modalities. Negative valence processing in the right hemisphere and emotional salience reduce susceptibility to these cueing effects.

### Conflict of interest statement

The authors declare that the research was conducted in the absence of any commercial or financial relationships that could be construed as a potential conflict of interest.
